# Macon Medical Society

**Published:** 1902-01

**Authors:** 


### MACON MEDICAL SOCIETY.

NOVEMBER 19.

At a meeting of the Macon Medical Society the following officers were elected: Dr. Carswell, President; Dr. McAfee, Vice-President; Dr. Stapler, Secretary; Dr. Ferguson, Reporter.

On motion, the regular order of business was suspended, and Dr. Ferguson read the following bills he was desirous to have enacted as laws, which met with the approval of the society. The substance of the bills is as follows:

The Macon Medical Society is preparing, through a committee from its membership, a bill providing that all coroners in Georgia shall be practicing physicians.

The society also calls for a reform in the manner of holding lunacy trials, and will present a bill embracing their substitute for the present law. They would have each lunacy candidate examined by three different physicians, to be appointed by the ordinary as a jury. Each of the physicians shall examine him on a separate day and then all three shall examine him at the same time, and make a verdict, the time consumed being not to exceed four days.

It is also proposed by the society to establish a school of science, the duty of which shall be to examine from time to time all drugs or proprietary medicines introduced into the State and require such manufacturer to distinctly set forth on the label of such proprietary medicine all of the constituent elements of said proprietary medicine and the respective proportion of each element, so as to be easily read by the public at large.

Also the society proposes that morphine, cocaine, opium, or any other narcotic or poisonous drug, as carbolic acid, nitric acid, sulphuric acid or any other poisonous acid or drug, shall not be sold to any one without a prescription from and over the signature of a reputable and licensed physician.

Drs. Ferguson, Barron and Carswell were appointed the committee to prepare bills covering these matters and to present them to the legislature. Dr. Ferguson is chairman of the committee, the propositions to be formulated in the bill having been submitted to the society by him.

The President appointed Drs. Ferguson, Carswell and Barron to consult and obtain legal advice and have the bills framed and placed upon the calendar of the legislature, so as to be in readiness for next session.

